# Topics of the Month

**Published:** 1893-02

**Authors:** 


### TOPICS OF THE MONTH.

THE biennial report of the Trustees of the Eastern Michigan Asylum for the Insane, at Pontiac, for the period ending June 30, 1892, is one of the most creditable reports of similar institutions that we have seen. The fire of December 26, 1891, which made such havoc, and so nearly destroyed the institution, is graphically depicted by photographic reproductions. The work of reconstruction began at once, and everything is in ample order again. Dr. C. B. Burr, the accomplished medical superintendent, makes a most elaborate report of his official work for the biennial, occupying seventy pages of the brochure. Considerable interest is added to Dr. Burr's report, from the fact that to the medical staff of the asylum is attached, as consulting gynecologist, Dr. W. P. Manton, who has made a number of abdominal sections for the relief of insane patients suffering from pelvic disease. In doing so, Dr. Manton has had the cordial support of the asylum management in both the business and medical departments. The result of this work has been lately given out to the professional world in a paper published by Dr. Manton in the November number of the *American Journal of Obstetrics*, which we advise every one interested to read. Dr. Rohé, Superintendent of the Maryland Institution, at Catonsville, has been pursuing this same work on a more extended scale, and his results are also given out in the same journal. Both these papers were read at the St. Louis meeting, September 1, 1892, of the American Association of Obstetricians and Gynecologists, and the Transactions of the Association for the year 1892 will contain both these papers, together with the discussions thereon. We predict that the work inaugurated by Drs. Manton and Rohé will lead to a reconstruction of opinions heretofore held by superintendents of asylums on the subject in question. There is need that every insane hospital should have attached to its medical staff a competent gynecologist, who is prepared by training and equipment to do abdominal surgery when necessary.

---

IN THE report of the Chief of the Bureau of Medicine and Surgery, U. S. Navy, for 1892, Dr. Albert L. Gihon, Medical Director, gives the present status of the U. S. Naval Hospital, at Brooklyn, up to the date of his relief from duty there. Dr. Gihon suggests the peculiar fitness of this hospital, which is one of the best appointed hospitals in New York, for the establishment of a "Naval Medical School of Practice," for the instruction in higher medicine of the young entrants in the medical corps. This would be a marked advance in the conduct of naval medical affairs, and evidently is one of the ever-growing needs of the service. It seems to us that Dr. Gihon's recommendation should be promptly adopted.

Medical Director Wales reports upon the growth of the Naval Museum of Hygiene, under his charge. This institution is one of the three great museums of this character in existence, and in the extent and practical value of its exhibits surpasses the other two at London and Berlin.

The report, altogether, is an interesting one, and will well repay a careful reading by all physicians who bear any relation to the naval medical service, either as members of the staff or interested observers of its work.

---

The time has passed when it is pertinent to discuss the propriety of warming street cars in Winter, either from a standpoint of hygiene, sanitation, or decency. There are no scientific arguments that can hold on the negative side of the question, neither are any such offered. The only opposition to the measure seems to come from a few sophomore editors of daily newspapers and the managers of the street railway companies. With these we have no issue, because we do not expect them to be scientific or unselfish. If there is any doubting mind on the question, either as related to sanitation or comfort, let him take an early morning ride from Main street to Black Rock on one of the delightfully pleasant and comfortably warm cars of the Niagara line, and on his return to Main street board a Cold Spring car going north. Upon his return to the center of business, we think he will vote for the cars that are provided with heaters by a large majority.

As we remarked at the outset, there is no discussion now as to the propriety of properly heating the street cars; the only question for debate, and that is chiefly on grounds of economics, is the method of providing heat for the same. But, let not our esteemed managers wait for a better system, but put in stoves at once, and when something more serviceable is devised, let that be adopted; in other words, let us show ourselves to be a progressive city in whatever contributes to the comfort, health, and prosperity of our citizens. Not a visitor from another city comes to us but that criticises, in no weak terms, our present system of street railway management.

---

Civil Service Examinations.—John B. Riley, Chief Examiner, under date of January 19, 1893, announces an open competitive examination of candidates for the positions of first assistant, junior assistant, and female physicians, and apothecaries in the State Hospital Service, will be held at the rooms of the New York Civil Service Commission, in the Capitol, Albany, on Tuesday, February 28th, at ten o'clock a. m.

Applicants for the position of first assistant must be graduates of a legally incorporated medical college. They must be not less than twenty-five years of age, and must have had three years'

experience as physician in a hospital for the insane. Applicants for the positions of female and junior assistant physicians must be graduates of a legally incorporated medical college, and must have had one year's experience in a hospital, or three years' experience in the general practice of medicine. Applicants for the position of apothecary must have a license from the State Board of Pharmacy. All applicants must be citizens, and residents of the State of New York during the year last past.

For application blanks and other information, address Clarence B. Angle, Secretary, New York Civil Service Commission, Albany, N. Y.

---

The Toledo *Medical Compend*, for December, 1892, which, by the way, arrived on the 12th day of January, 1893, contains an editorial on the subject of advertisers and advertisements that is as remarkable for its statements as it is misleading. No doubt, advertisers in medical journals benefit the medical profession in divers and various ways; we need not discuss that question now. But when a medical journal that holds itself up to be an exemplar of ethical propriety and an expounder of the code as found in the by-laws of the American Medical Association, asserts that a strictly scientific man is not popular in this age of the world, and that to be scientific does not bring financial fruit, it is about time that some effort was made to prevent such dangerous doctrine from spreading itself out among the young candidates for the degree of doctor of medicine.

---

Mr. E. B. Treat, medical publisher, of New York, announces that he has in press for early publication the International Medical Annual for 1893. This is the eleventh yearly issue of this valuable work, and a glance at the prospectus gives promise that it will be superior to any of its predecessors. The original corps of thirty-eight specialists are retained as editors, and these have been wisely chosen from among the more prominent physicians and surgeons of America, Great Britain, and the Continent of Europe.

In this book will be found, arranged in a practical manner for ready reference, whatever is worth preserving of the year's medical literature, together with a number of papers written especially for the Annual, and it will contain over 6,000 references to diseases and their remedies. It is illustrated with a number of instructive cuts, in black and colors, that are serviceable in explaining the text. The price of the book, $2.75, is very reasonable, and ought to put it into the hands of every practitioner in this country who considers himself a progressive physician.

---

The city newspapers are entitled to the thanks of the profession for the marked courtesy with which they treated the Medical Society of the County of Erie at its last meeting. Generally speaking, the flaming headlines have indicated something other than the text when notices of doctors' meetings have appeared in the newspapers. We observed that the *Buffalo Commercial* published the dignified headline, "Our Doctors Meet," which was certainly everything that could be desired in that direction; and so with other city dailies that were quite as considerate with their display of types, if not as apt. This all shows an increase of respect for a profession that certainly has a right to expect fair treatment at the hands of the daily press.

---

The *Medical Bulletin* is the title of a weekly medical newspaper published by the Kauffman Medical Publishing Company, of Chicago. It is edited by Dr. M. W. Kauffman, and is intended to cover the field of a weekly newspaper devoted entirely to medical affairs. In other words, it will not publish long scientific dissertations, but only items of medical news, and will be especially devoted to the interest of the physicians during the Columbian Exposition. By registering with the *Bulletin*, physicians will have the advantage of obtaining every convenience during their sojourn in Chicago at a reasonable cost, and thus avoid the possibility of extortion. This enterprise on the part of Dr. Kauffman is worthy of support.

---

At the January meeting of the Pittsburg Obstetrical Society, Dr. John Milton Duff reported having performed a pubeotomy on Mrs. Deitrich Smith. Mother and child were both doing well on the eighteenth day.

